# Mastitis-related *Staphylococcus aureus*-derived extracellular vesicles induce a pro-inflammatory response in bovine monocyte-derived macrophages

**DOI:** 10.1038/s41598-025-90466-6

**Published:** 2025-02-19

**Authors:** Mara D. Saenz-de-Juano, Giulia Silvestrelli, Samuel Buri, Léa V. Zinsli, Mathias Schmelcher, Susanne E. Ulbrich

**Affiliations:** 1 Animal Physiology, Institute of Agricultural Sciences, ETH Zurich, Zurich, 8092 Switzerland; 2 Institute of Food, Nutrition and Health, ETH Zurich, Zurich, 8092 Switzerland; 3 ZHAW School of Life Sciences and Facility Management, Fachstelle Biochemie und Bioanalytik, Einsiedlerstrasse 31, Wädenswil, 8820 Switzerland; 4 Institute of Science and Technology Austria, Klosterneuburg, 3400 Austria

**Keywords:** Infection, Animal physiology

## Abstract

*Staphylococcus aureus* (*S. aureus*) is one of the most common causative agents of mammary gland infection and mastitis, but the specific role of *S. aureus*-derived extracellular vesicles (SaEVs) in mastitis has been poorly studied to date. Here, we aimed to investigate the response of bovine monocyte-derived macrophages (boMdM) to SaEVs of the genotype B (GTB) mastitis-related strain M5512B. Specifically, we evaluated the effects on the actin cytoskeleton, gene expression, and the SaEV proteomic cargo. Furthermore, we assessed to what extent the cellular and molecular response of boMdM to SaEVs differed from peripheral mononuclear blood cells (PBMCs) used for in vitro derivation of the former. We observed that SaEVs induced morphological changes in boMdM, leading to a pro-inflammatory and pyroptosis-related increased gene expression. Additionally, our study revealed that boMdM and PBMCs exhibited stimulus-specific differing responses. The proteomic analysis of SaEVs identified clusters of proteins related to virulence and antibiotic resistance, supporting the theory that *S. aureus* might use EVs to evade host defences and colonize the mammary gland. Our results bring new insights into how SaEVs might impact the host during an *S. aureus* infection, which can be useful for future *S. aureus* vaccine development.

**Supplementary Information:**

The online version contains supplementary material available at 10.1038/s41598-025-90466-6.

## Introduction

Mastitis, the inflammation of the mammary gland, can develop when the host’s innate and adaptive defences fail to prevent the invasion and establishment of microorganisms in the gland tissue^1,2^. Apart from a significant reduction in milk yield and quality, mastitis in dairy farms imposes further costs, such as an increased workload, discarded milk, reduced suitability for cheesing, veterinary costs and antibiotic treatment. For that reason, mastitis is one of the costliest diseases in dairy farming worldwide and a leading cause of premature end of cows’ production periods and increased antimicrobial use^3,4^.

The Gram-positive bacteria *Staphylococcus aureus* (*S. aureus)* is one of the main causative agents for mastitis^2^. Often, *S. aureus* causes an asymptomatic/subclinical form of mastitis that is not readily detectable to either farmer or veterinarian and can easily be transmitted to other animals in the herd during the milking routine^5^. Subclinical mastitis can lead to chronic, possibly lifelong disease^1^. Furthermore, *S. aureus* expresses a set of antimicrobial resistance genes^6,7^, which, together with its ability to form biofilms and live inside the mammary gland cells, contributes to the major problem of therapeutic antibiotic failure^2^. The epidemiology of *S. aureus* markedly depends on the particular genotype^8,9^. In particular, *S. aureus* genotype B (GTB) is of primary concern because it is related to high contagiousness and pathogenicity^8,10–12^. *S. aureus* GTB has been associated with intramammary infections and was found to be highly abundant in raw milk cheese, risking enterotoxin intoxication in humans^13^.

It has been shown that Gram-positive bacteria, including *S. aureus*, can produce vesicular bodies carrying toxins and enzymes^14–17^. Bacterial EVs play crucial roles in bacterial physiology, pathogenic processes, environmental adaptation and infections^18^. The proteins and nucleic acids encapsulated within *S. aureus* extracellular vesicles (SaEVs) are protected from degradation or neutralisation by the host and, therefore, play an important role during pathogenesis^19^. In mice, it was demonstrated in vivo that SaEVs can cause a local inflammatory reaction in the mammary gland^20^ and induce apoptosis of lung cells^16^ and arthritis^21^. In vitro, SaEVs induce a dose-dependent pro-inflammatory response in peritoneal macrophages and splenocytes^21^ that is mediated by lipoproteins and via Toll-like receptor 2-dependent mechanisms^21^.

In the context of mastitis, bacterial EVs intensify the inflammatory response and contribute to infection within the mammary gland^20^. However, the pro-inflammatory effect of SaEVs on the bovine udder is unknown since in vivo studies in cows assessing the mammary gland response to SaEVs are still lacking. We have previously shown that primary mammary epithelial cells of cattle minimally respond to SaEVs, which could explain the high capacity of *S. aureus* to affect the mammary epithelial cells effectively^22^. However, the effect of SaEVs on the innate immune system of the cow udder is still unknown. Monocytes and their tissue derivatives, such as macrophages and certain dendritic cell subpopulations, play crucial roles in nearly all stages of inflammation^23^. Macrophages interact with *S. aureus*, especially in the early stages, to control and restrict the initial bacterial inoculum. They are essential to contain and resolve *S. aureus* infections. However, in its defence, *S. aureus* has developed numerous tactics to evade and manipulate host cells for its benefit^24^. Among the self-defence strategies, *S.aureus* can alter the actin cytoskeleton of the host cell, specifically by disaggregating cell-cell contacts^25^ and focal adhesions^26^ and consequently damaging the epithelial barriers.

We hypothesised that *S. aureus* uses SaEVs to favour the establishment of chronic subclinical infections by dampening the immune-related activities and escaping the innate host response. Thus, in this study, we aimed to investigate the effect of SaEVs on immune cells. For this reason, we evaluated the macrophage morphological and molecular gene expression response using bovine monocyte-derived macrophages (boMdM) and compared it to the peripheral mononuclear blood cells (PBMCs) of origin.

## Results

### M5512B SaEVs isolation and characterization

**Fig. 1:**
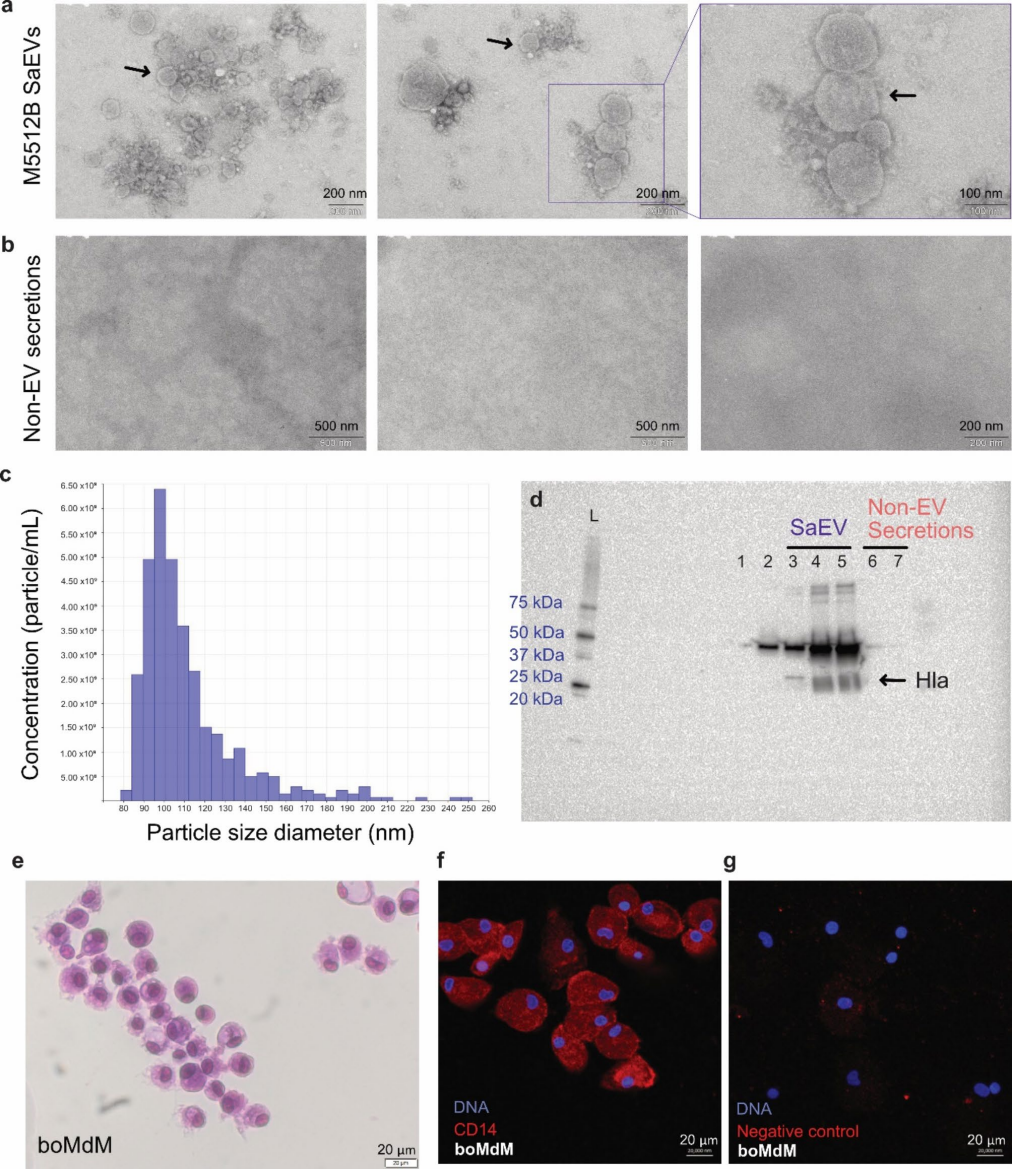
Successful isolation of *S. aureus* M5512B extracellular vesicles (SaEVs) and in vitro generation of bovine monocytes derived macrophages (boMdM). **(a)** TEM images showing heterogenic populations of SaEVs. Black arrows point to representative SaEV structures. **(b)** TEM images confirm the absence of SaEVs in the EV-depleted supernatant (non-EV secretions). **(c)** Particle concentration and size distribution of SaEVs used for the in vitro stimulation. **(d)** Full WB membrane shows alpha-toxin (Hla) in SaEVs (3–5) but not in non-EV secretions (6,7), TSB medium without *S. aureus* (1), or non-processed *S. aureus* medium (2). In samples 2–5, a strong band around 50 kDa is observable, which might indicate the presence of an immunoglobulin-binding protein that binds to the Fc regions of both the primary and secondary antibodies^22^. L: protein size ladder. **(e)** Diff-Quik staining of the in vitro derived boMdM used for the experiments showed the typical flattened, adherent shape with an amoeboid appearance macrophage morphology. **(f)** Confocal imaging confirmed that boMdM were positively stained with CD14, commonly considered a macrophage marker. **(g)** Negative control staining included only the secondary antibody.

The isolated SaEVs from the M5512B *S. aureus* strain were characterized using transmission electron microscopy (TEM), tunable resistive pulse sensing (TRPS), and western blot (WB). The TEM images showed cup-shaped intact vesicles of different sizes in the SaEV pellet (Fig. 1a) and the absence of vesicles and aggregates in the non-EV secretions (Fig. 1b). The isolated particles had a diameter in the 80–250 nm range, with the higher population being around 100 nm (Fig. 1c). We then confirmed by WB the presence of the *S. aureus* toxin alpha-hemolysin (Hla) in our SaEVs extract but not in the non-EV secretions (Fig. 1d).

### Bovine monocyte-derived macrophages (boMdMs) characterization

The adherence-based method was employed to differentiate attached monocytes from the PBMC pool into macrophages over a 7-day culture period. Most attached cells changed to a typical macrophage morphology (Fig. 1e). In addition, we observed that most of the cells were CD14-positive (Fig. 1f, g). CD14 is an endotoxin receptor typical of monocytes and tissue macrophages expected to be present in boMdM (Günther et al., 2016).

### M5512B *S. aureus* secretions induce changes in the actin cytoskeleton of boMdM

**Fig. 2:**
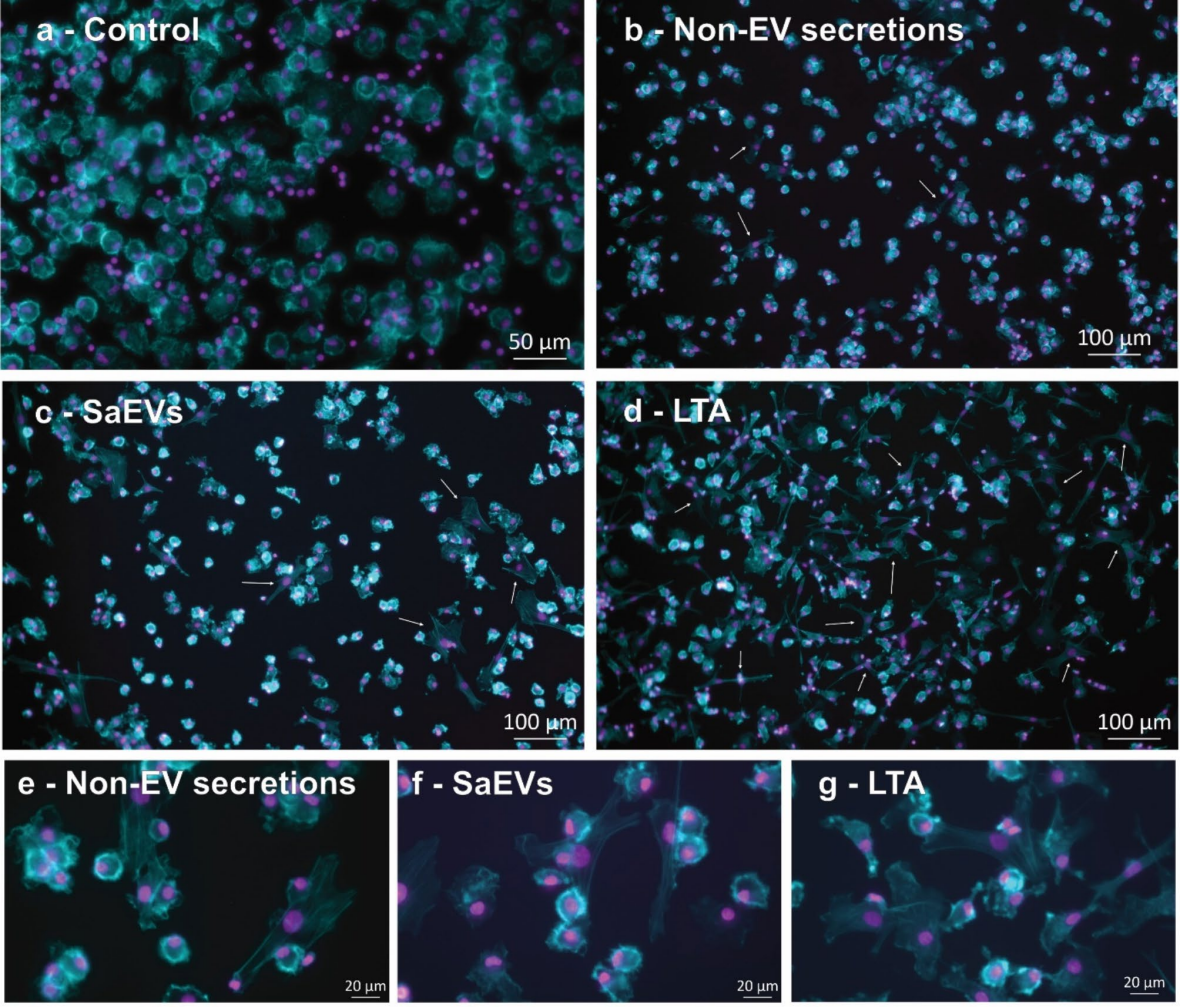
Fluorescence microscopy images showing the different morphologies of boMdM 24 h after stimulation with **(a)** PBS (Control), **(b)** non-EV secretions, **(c)** SaEVs, and **(d)** LTA. Different magnifications illustrate the typical morphology of non-stimulated cells, the abundance of morphological changes, and specific cytoplasmic changes in response to stimuli. Actin cytoskeleton was stained with phalloidin (blue), and DNA was stained in DAPI (purple). White arrows point to those boMdM with morphological changes. **(e-g)** Additional fluorescence microscopy images were acquired at a higher magnitude to appreciate the cytoplasmatic changes in boMdM.

Since the rearrangement of the cytoskeleton in the host cells is a dominant feature of *S. aureus* infection, we evaluated the actin cytoskeleton using phalloidin staining 24 h after stimulation with SaEVs, non-EV secretion, or lipoteichoic acid (LTA). We observed how exposure to all *S. aureus* secretions induced morphological changes in boMdM (Fig. 2a-g). More cells changed morphology after LTA exposure as compared to SaEVs followed by non-EV secretions. No additional signals were detected in the DNA channel beyond the nuclear region, ruling out the presence of macrophage extracellular traps (MET) after 24 h of culture.

### Pro-inflammatory response of boMdM to M5512B SaEVs

**Fig. 3:**
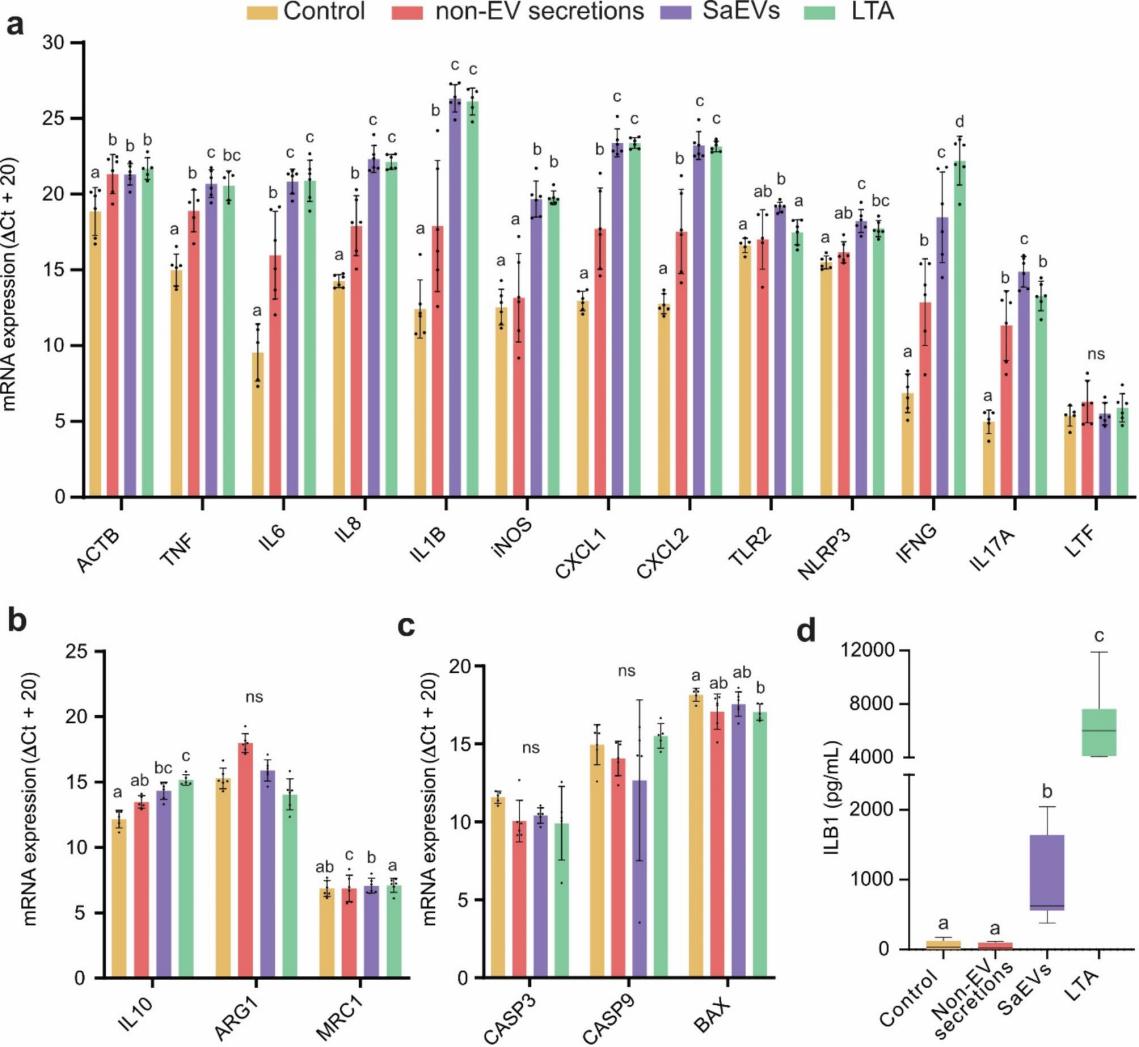
Effect of M5512B SaEVs on bovine Monocytes derived Macrophages (boMdM). Relative gene expression of pro-inflammatory genes **(a)**, anti-inflammatory genes **(b)**, and apoptosis-related genes **(c)** in boMdM 24 h after stimulation with PBS (control), non-EV secretion, SaEV, or LTA. Bar charts show the mean and standard deviation. Groups with different letters are statistically different from each other at a significance level of *P* < 0.05. “ns” denotes no significant differences. **(d)** Interleukin 1B protein release in boMdM 24 h after stimulation with PBS (control), non-EV secretion, SaEVs, or LTA.

Incubation with SaEVs significantly upregulated several typical pro-inflammatory genes, including interleukin 1 beta (IL1B), interleukin 6 (IL6), interleukin 8 (IL8), tumor necrosis factor (TNF), interferon-gamma (IFNG), inducible nitric oxide synthase (iNOS), C-X-C motif chemokine ligand 1 (CXCL1), and C-X-C motif chemokine ligand 2 (CXCL2) (Fig. 3a). This upregulation was comparable to LTA and was higher than that caused by non-EV secretions. Additionally, SaEVs led to a significantly increased expression of interleukin 17 A (IL17A), showing a more pronounced effect than either non-EV secretions or LTA (Fig. 3a). Also the beta-actin gene (ACTB) expression showed increased transcript abundance following all types of stimulation (Fig. 3a).

The expression of lactotransferrin (LTF), which can act as either a pro-inflammatory or anti-inflammatory gene, did not change after incubation with SaEVs (Fig. 3a). Similarly, the transcript abundance of the typical anti-inflammatory genes mannose receptor C-type 1 (MRC1) and arginase 1 (ARG1) remained unchanged. In contrast, the expression of interleukin 10 (IL10) was significantly upregulated, similar to the effect seen with exposure to lipoteichoic acid (LTA) (Fig. 3b).

### M5512B SaEVs are likely to initiate pyroptosis instead of apoptosis in boMdM

Incubation with SaEVs and non-EV secretions for 24 h did not change the apoptosis-related gene expression of caspase 3 (CASP3), caspase 9 (CASP9), and BCL2-associated X protein (BAX) (Fig. 3c). However, in the case of SaEVs, we observed a significant upregulation of the toll-like receptor 2 (TLR2) gene and the inflammasome-related gene NLR family pyrin domain containing 3 (NLRP3). The gene expression changes were accompanied by increased cytokine interleukin 1 beta (IL1B) protein release into the medium, which was not observed in neither control nor non-EV secretions groups (Fig. 3d).

### Differential gene expression response between boMdM and peripheral blood mononuclear cells (PBMCs) to M5512B SaEVs and non-EV secretions

**Fig. 4:**
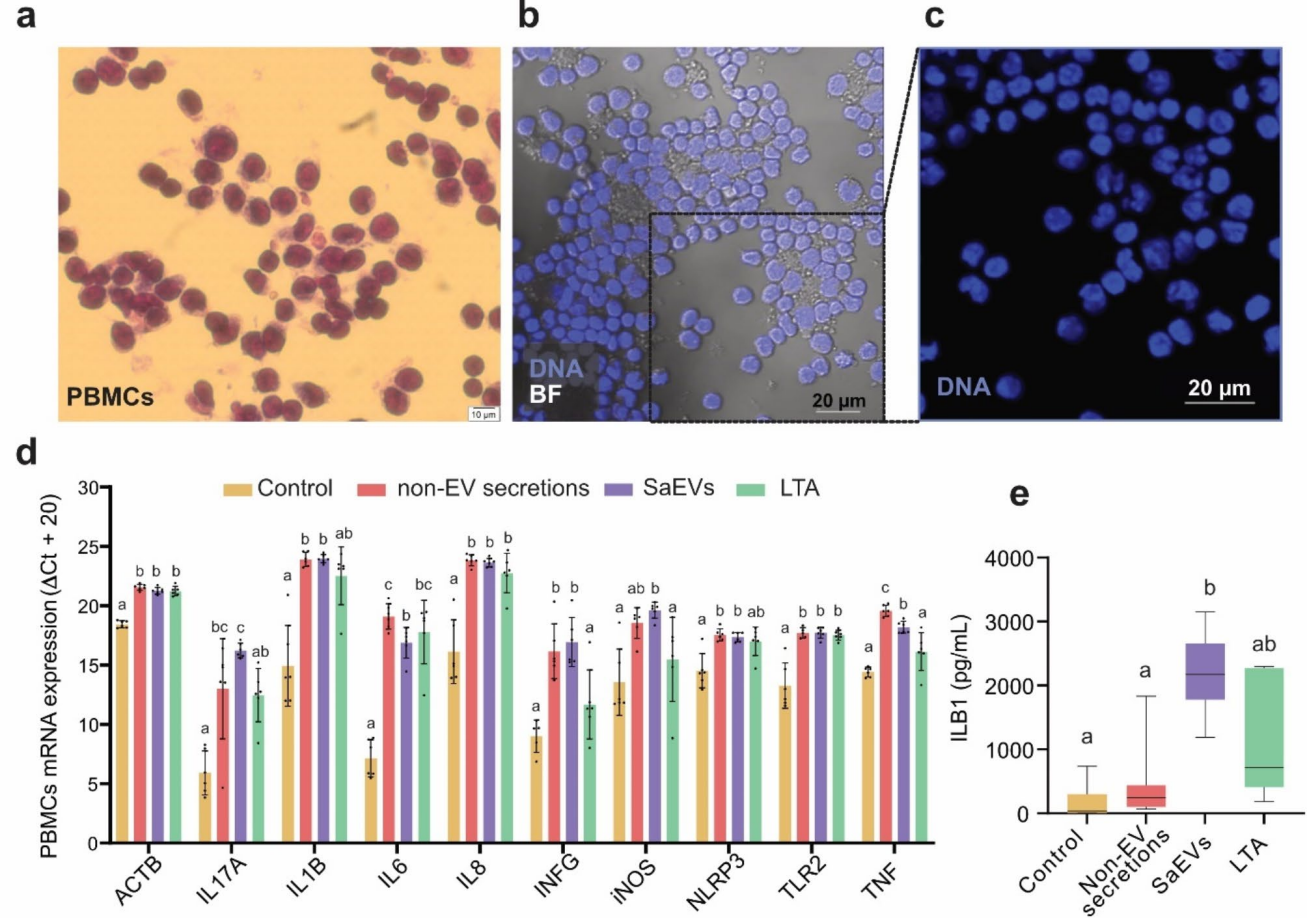
Effect of M5512B SaEVs on bovine peripheral blood mononuclear cells (PBMCs). **(a)** Diff-Quik staining of isolated bovine PBMCs. Scale bar: 10 μm. **(b)** Merged image of DNA staining (blue) and brightfield (BF, grey) imaging, showing the different nuclear compositions of the PBMCs. **(c)** Enlarged image illustrating that the PBMC composition includes lymphocytes (round-shaped nuclei, red arrow) and monocytes (kidney-shaped nuclei, white arrow). **(d)** Relative gene expression of pro-inflammatory genes in PBMCs 24 h after stimulation with PBS (control), non-EV secretion, SaEV, or LTA. Bar charts show the mean and standard deviation. Groups with different letters are statistically different from each other at a significance level of *P* < 0.05. **(e)** Interleukin 1B protein release in PBMCs 24 h after stimulation with PBS (control), non-EV secretion, SaEVs, or LTA.

To evaluate the specificity of the response of boMdM, we stimulated bovine PBMCs using the same *S.aureus* agents as with boMdM. Isolated PBMCs showed a different morphology than boMdM (Fig. 4a). Specifically, bovine PBMCs include a large number of lymphocytes (T cells, B cells, and Natural Killer cells), characterized by small, round nuclei with densely packed chromatin, giving a strong and uniform DAPI signal, and monocytes, which often display a larger kidney-shaped nucleus with more dispersed chromatin (Fig. 4b, c).

In the PBMCs, the gene expression of ACTB, IL1B, IL6, IL8, IFNG, iNOS, TNF, TLR2, IL17A, and NLRP3 was upregulated 24 h after SaEV exposure. However, unlike in boMdM, the non-EV secretions also induced a higher or similar upregulation (Fig. 4d). We also observed that LTA stimulation did not affect the expression of IL1B, IL17A, INFG, iNOS, TNF, or NLRP3 or the release of IL1B protein (Fig. 4e). Indeed, in PBMCs, the release of ILB1 was only significantly increased after SaEVs stimulation.

### Proteomic analysis of M5512B SaEVs and non-EV secretions

**Fig. 5:**
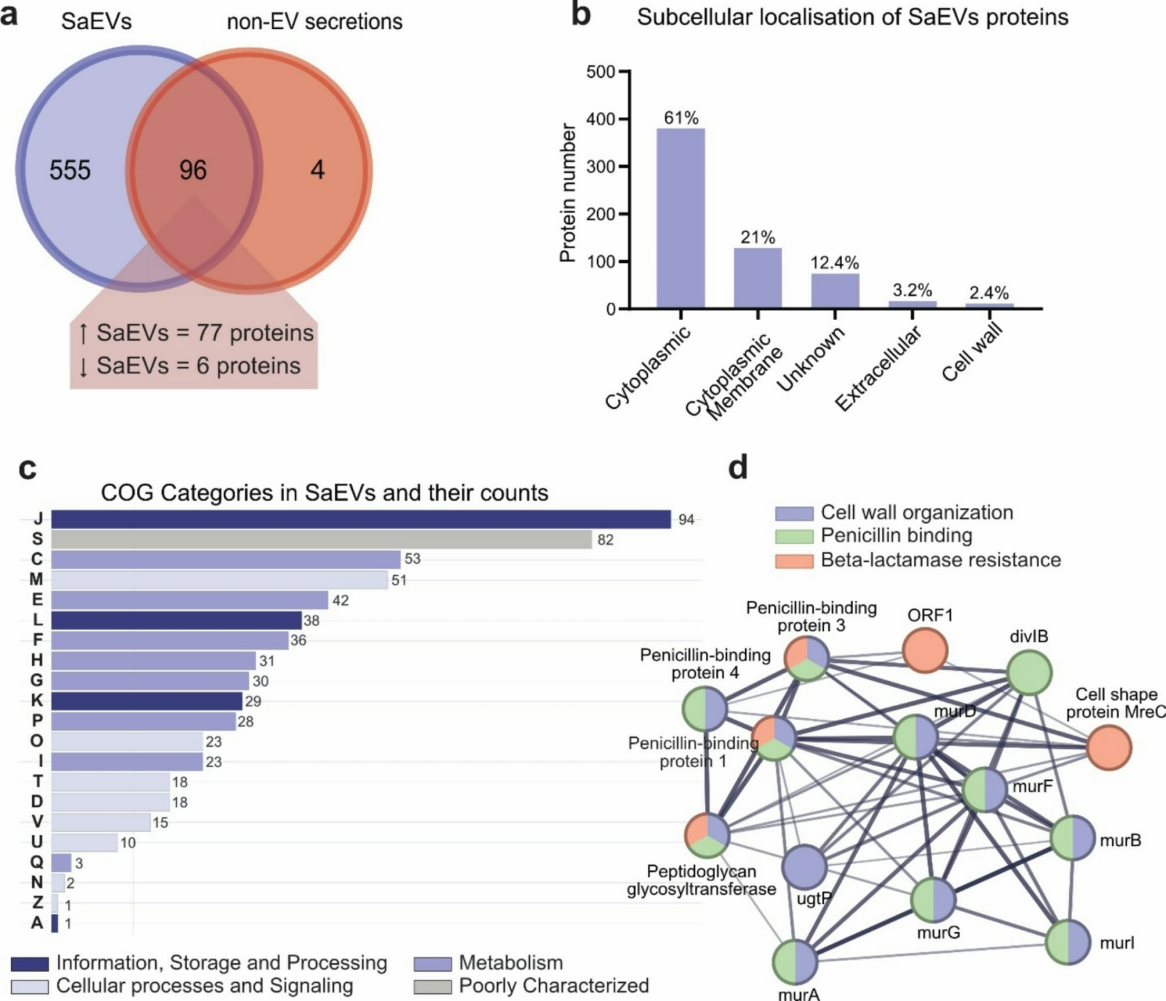
**(a)** The Venn diagram shows unique and shared proteins identified in SaEVs and non-EV secretions. **(b)** Bar chart showing the different subcellular locations and percentages of the proteins identified in SaEVs. **(c)** The Clusters of Orthologous Groups (COGs) categories analysis. Adjacent numbers on the bar charts denote the total proteins identified per category. A: RNA processing and modification; C: Energy production and conversion; D: Cell cycle control, cell division, and chromosome partitioning; E: Amino acid transport and metabolism; F: Nucleotide transport and metabolism; G: Carbohydrate transport and metabolism; H: Coenzyme transport and metabolism; I: Lipid transport and metabolism; J: Translation, ribosomal structure, and biogenesis; K: Transcription; L: Replication, recombination, and repair; M: Cell wall/membrane/envelope biogenesis; N: Cell motility; O: Post-translational modification, protein turnover, and chaperones; P: Inorganic ion transport and metabolism; Q: Secondary metabolites biosynthesis, transport, and catabolism; S: Function unknown; T: Signal transduction mechanisms; U: Intracellular trafficking, secretion, and vesicular transport; V: Defense mechanisms; Z: Cytoskeleton. **(d)** Protein-protein interaction of cluster 7 (Supplementary Tables 3, 14 proteins) showing the most significant terms: cell wall organisation (purple), penicillin-binding (green), and beta-lactamase resistance (red).

To understand the origin of the morphological and gene expression changes in boMdM and PBMCs, we performed a proteomic analysis of the SaEVs and non-EV secretions used for the in vitro stimulation. The protein identification analysis revealed 655 unique proteins. From these, 555 proteins were present only in SaEVs, 4 proteins were present only in non-EV secretions, and 96 proteins were shared between both (Fig. 5a, Supplementary Table 1). From the shared proteins between SaEVs and non-EV secretions, 77 proteins were more abundant (fold change > 2) in SaEVs, while only 6 proteins were more abundant (fold change < 0.5) in non-EV secretions (Supplementary Table 1). The protein with the highest count for SaEVs and non-EV secretions was the Immunoglobulin-binding protein Sbi, with 586 counts in SaEVs and 45 in non-EV secretions (Supplementary Table 1). The Sbi is an *S. aureus* immunoglobulin-binding protein that inhibits opsonisation and phagocytosis by attaching to the Fc region of the antibodies. This protein is approximately 60 kDa in size, which might correspond to the prominent nonspecific band we observed in the WB, showing a higher intensity in SaEVs compared to non-EV secretions (Fig. 1d). When analysing the proteins uniquely present or more abundant in non-EV secretions, the predicted location was cytoplasmic or membrane-associated (Fig. 5b, Supplementary Table 2). The highest proportion of proteins in SaEVs were related to cellular and metabolic processes (Supplementary Table 3). In particular, the COG category with a higher count was “Translation, ribosomal structure, and biogenesis”, followed by “Energy production and conversion” and “Cell wall/membrane/envelope biogenesis” (Fig. 5c, Supplementary Table 4). We further used protein-to-protein interaction analyses to evaluate the potential links between those more abundant proteins in SaEVs. We performed a cluster analysis and obtained 8 clusters with more than 10 proteins (Supplementary Table 5). The first 5 clusters, including 80 to 22 proteins, had a structural function such as translation, ribosome biogenesis, metabolic processes and DNA replication. Cluster 6 was related to peptidoglycan biosynthesis, the major structural component of the *S. aureus* cell wall. Interestingly, in Cluster 7, with 14 proteins included, the most significant terms were cell wall organisation, penicillin-binding, and beta-lactamase resistance (Fig. 5d). Finally, in Cluster 8, most enriched categories were related to the stress response. We could also observe that some of the proteins isolated within SaEVs (Table 1) were related to pathogenesis or virulence (Uniprot-Keywords KW-0843) or included in the Virulence Factor Database (VFDB). Moreover, some virulence-associated proteins were also classified as core pathogenic proteins from several *S. aureus* strains isolated from cattle or humans^27^.

**Table 1:** Selection of virulent proteins included in M5512B *Staphylococcus aureus*-derived extracellular vesicles.

Gene Symbol	Gene Name	Uniprot access num.	Biological process	Molecular Function	Conserved EV core protein
agrB	Accessory gene regulator protein B	Q2FWM7	Proteolysis, quorum sensing	Peptidase activity	
alt	Bifunctional autolysin	Q2FZK7	Peptidoglycan catabolic activity	Amidase activity	yes
clfB	Clumping factor B	Q2FUY2	Virulence		
clpC	ATP-dependent Clp protease ATP-binding subunit ClpC	Q2G0P5	Response to stress, virulence	ATP binding	
coa	Coagulase	Q2G1D3			
crtP	4,4’-diaponeurosporene oxygenase	Q2FV57	Carotenoid biosynthesis, virulence		
cvfB	Conserved virulence factor B	Q2FYP3			
dltD	Protein DltD	Q2FZW3	lipoteichoic acid biosynthetic process		yes
eno	Enolase	Q2G028	Glycolysis, virulence	Lyase	yes
epbS	Elastin-binding protein	Q2FYF1			yes
fmtA	Teichoic acid D-alanine hydrolase	Q2FZK3	response to antibiotic	hydrolase activity	
ftnA	Bacterial non-heme ferritin	Q2FWZ8	Iron storage	Oxidoreductase	yes
ftsH	ATP-dependent zinc metalloprotease FtsH	Q2G0R0	proteolysis	Metalloprotease	yes
gapA1	Glyceraldehyde-3-phosphate dehydrogenase	Q2G032	glucose metabolic process	Oxidoreductase	yes
hld	Delta-hemolysin	Q2FWM8	Hemolysis, virulence	Toxin activity	yes
hlgB	Gamma-hemolysin component B	Q2FVK1	Hemolysis, virulence	Toxin activity	
hlgC	Gamma-hemolysin component C	Q2FVK2	Hemolysis, virulence	Toxin activity	
hly	Alpha-hemolysin	Q2G1 × 0	Hemolysis, virulence	Toxin activity	
htrA	Serine protease HtrA-like	Q2FXJ6	proteolysis	Serine protease	yes
SAOUHSC_02241	leukocidin-like protein 1	Q2FWP0	cytolylisis in another organism		
SAOUHSC_02243	leukocidin-like protein 2	Q2FWN9	cytolylisis in another organism		
ltaS	Lipoteichoic acid synthase	Q2G093	cell wall organization	metal ion binding	yes
lytM	Glycyl-glycine endopeptidase LytM	O33599	Proteolysis, cell wall organization	metalloendopeptidase activity	
mgrA	HTH-type transcriptional regulator MgrA	Q2G0B1	Response to stress	DNA-binding	
mprF	Phosphatidylglycerol lysyltransferase	Q2G2M2	response to antibiotic	aminoacyltransferase activity	
pbp1	Penicillin-binding protein 1	Q2FZ94	cell wall organization	penicillin binding	
pbp3	Penicillin-binding protein 3	Q2FY21	cell wall organization	penicillin binding	yes
pbp4	Penicillin-binding protein 4	Q2G2 × 6	Proteolysis, response to antibiotic	beta-lactamase activity	yes
psmA1	Phenol-soluble modulin alpha 1 peptide	P0C7Y1	killing of cells of another organism		yes
psmA2	Phenol-soluble modulin alpha 2 peptide	P0C7Z3	killing of cells of another organism		yes
psmA4	Phenol-soluble modulin alpha 4 peptide	P0C818	killing of cells of another organism		yes
rny	Ribonuclease Y	Q2FZ08	mRNA catabolic process	RNA endonuclease activity	
saeS	Histidine protein kinase SaeS	Q2G2U1	Virulence	phosphorelay sensor kinase activity	
sarA	Transcriptional regulator SarA	Q2G2U9	Transcription regulation	DNA-binding	
sarS	HTH-type transcriptional regulator SarS	Q2G1N7	response to stress	DNA binding	
sarV	HTH-type transcriptional regulator SarV	Q2FVY9	Virulence	DNA binding	
sarX	HTH-type transcriptional regulator SarX	Q2G0D1	Virulence	DNA binding	
sbi	Immunoglobulin-binding protein Sbi	Q2FVK5	Virulence	IgG binding	yes
sdrC	Serine-aspartate repeat-containing protein C	Q2G0L5	cell adhesion		
secA1	Protein translocase subunit SecA 1	O06446	Protein transport	metal ion binding	yes
selX	Enterotoxin-like toxin X	Q2G107			
sl11	Staphylococcal superantigen-like 11	Q2G0 × 4	Virulence		
sle1	N-acetylmuramoyl-L-alanine amidase sle1	Q2G0U9	killing of cells of another organism	lytic endotransglycosylase activity	
srtA	Sortase A	Q2FV99	Proteolysis	metal ion binding	yes
ssaA	Staphylococcal secretory antigen SsaA	Q2FV55	Virulence		
ssaA2	Staphylococcal secretory antigen ssaA2	Q2G2J2	Virulence	hydrolase activity	yes
sspP	Staphopain A	Q2G2R8	Proteolysis, virulence	cysteine-type peptidase activity	
tcaA	Membrane-associated protein TcaA	Q2G2I1	response to antibiotic	metal ion binding	yes
traP	Signal transduction protein TRAP	Q2G2F3	Virulence		
tuf	Elongation factor Tu	Q2G0N0	translational elongation	GTP-binding	yes
vraS	Sensor protein VraS	Q2FX08	Two-component regulatory system	protein dimerization activity	yes

## Discussion

Monocytes are typically purified from PBMCs based on their adhesive properties or using magnetic-activated cell sorting (MACS)^23^. We opted for the adhesive method because it is simpler and does not require complex equipment. However, this method does not allow for the specific isolation of monocyte subsets (like the CD14 + fraction) with high-purity^23^. Therefore, we stained the derived boMdM with a CD14-specific antibody to verify that most of the cells used in the experiment were CD14-positive. Using cellular component staining, we also observed morphological differences between the in vitro-derived boMdM and their PBMCs of origin.

We isolated EVs from a mastitis-related strain, M5512B, which we previously named M5512VL^22^. We changed the name to acknowledge that it is a genotype B (GTB) strain. GTB and its variants form a genotypic cluster named CLB, which is almost exclusively associated with clonal complex 8 (CC8)^28^. Specifically, the M5512B strain carries the genomic island vSaβ type II, which contains several virulence-associated and pathogenic genes^29^. We selected this *S. aureus* genotype because it has been linked to a high prevalence of intramammary infections within herds, especially in Central Europe^30^. Additionally, it has been reported to release virulence factors that evade the immune response and favour the establishment of chronic and subclinical infections^30^. Whole-genome sequencing data of the M5512B strain is available in the NCBI database under the reference SZZF00000000 ^30^. Since the secretome of *S. aureu*s is very extended and not limited to EVs^19^, we included the same protein concentration of non-EV secretions as a control to assess the specificity of the effect of SaEVs stimulation and LTA as a positive control to ensure the responsiveness of our cells.

Alpha-hemolysin (Hla) was selected because its gene is present in the M5512B strain (located in contig00005, reference SZZF01000005.1) and has been reported as one of the most toxic pore-forming proteins found in *S. aureus* EVs^14^. Additionally, we have previously used Hla in our research^22^, obtaining consistent and reliable results with Western blot analysis. In the current study, its presence was further confirmed through the proteomic analysis of SaEVs.

Macrophages exert their immune function by rearranging the actin cytoskeleton, which is essential for migration^31^, phagocytosis of the invading pathogens^32^ and antigen presentation^33^. Immune stimulation can also upregulate the actin mRNA levels of macrophages^34^. On the other hand, bacteria developed numerous toxins and effectors acting in the absence of the bacteria itself. These effectors target the actin cytoskeleton to dampen the immune activity and to hijack the multitude of functions of the cytoskeleton for their own advantage^35^. Toxins such as Hla can induce rearrangements of the actin cytoskeleton in macrophages and epithelial cells^36^, which *S. aureus* uses to invade the host cells and evade detection and destruction by the host immune system^25^. We observed that all types of *S.aureus* stimuli upregulated actin gene expression and changed boMdM morphology. However, in the latter case, we cannot discern the contribution of regular macrophage immune function from an *S. aureus*-mediated cytoskeletal rearrangement.

To study the effect of SaEVs on boMdM gene expression, we included pro- and anti-inflammatory genes in the analysis. We observed that in boMdM, SaEVs induced a strong pro-inflammatory response of IL1B, IL6, IL8, iNOS, TNF, TLR2, CXCL1, and CXCL2, similar to the LTA and higher than the one produced by the non-EV secretions. On the contrary, the transcriptional abundance of the anti-inflammatory genes ARG1 or MRC1 was not changed. The expression of IL10 was significantly upregulated but to a lesser extent than other pro-inflammatory cytokines. The simpler composition in non-EV secretions compared to SaEVs may account for the attenuated immune response.

It has been shown that the reaction of boMdM to heat-killed *S. aureus* peaks at 3 h and decreases by 24 h, returning to homeostasis^37^. However, in our experiment, the pro-inflammatory upregulation of these genes remained active 24 h after stimulating with SaEVs. This was similar to what was observed in murine peritoneal macrophages^21^. Macrophages are professional antigen-presenting cells that can get activated by several bacterial epitopes^21,37^. Since SaEVs concentrate many cell membrane proteins and toxins, they can activate the macrophage pro-inflammatory response, even in the absence of the bacteria. This, together with the fact that SaEVs can also be released during bacterial cell lysis^19^, might help *S. aureus* to prolong the inflammatory process and cause tissue damage even after the bacteria are no longer alive.

While both boMdM and PBMCs exhibited significant upregulation of pro-inflammatory genes, the response in PBMCs was generally more pronounced. Moreover, SaEVs and non-EV secretions did not display pronounced differences in response. This result highlights the cell type-specific responses of PBMCs compared to boMdM and shows the inherent differences in sensitivity and function between the immune cells to *S. aureus* infections.

Our findings indicate that SaEVs preferentially activate pyroptosis rather than apoptosis in boMdM. Pyroptosis is an inflammatory form of cell death often triggered by bacterial infections that can act as a double-edged sword by eliminating infected cells but contributing to tissue damage and inflammation and potentially leading to detrimental effects on the host^38^. Pyroptosis initiation was evidenced by the significant upregulation of TLR2 and NLRP3 genes and increased release of IL1B. The lack of upregulation of apoptotic-related genes such as CASP3, CASP9, and BAX in boMdM further supports the selective activation of pyroptosis. The upregulation in TLR2 gene expression was significantly higher in SaEVs than in LTA. These results agree with previous reports in which chemically synthesized LTA did not activate TLR2 signalling^39,40^. In addition, SaEVs’ activation of TLR2 and cytokine transcription correlates with the presence of lipoproteins^41^, which are also included in the proteins identified in SaEVs.

The toxins we identified in our SaEVs might serve as a mechanism for *S. aureus* to induce inflammation, activate cell death, and promote its survival and dissemination within the host. Previous proteome analyses of EVs of different *S. aureus* strains revealed a content highly enriched in specific proteins responsible for transport, virulence, and pathological functions. Therefore, a specific sorting of proteins into EVs was suggested, named *S. aureus* core EV proteome^27^. Our proteomic analysis revealed that the cargo of SaEVs from the genotype B M5512B strain was similar to the *S. aureus* core EV proteome, mostly composed of cytoplasmatic proteins, followed by membrane proteins. Additionally, the majority of the proteins also belonged to the “translation, ribosomal structure, and biogenesis” group of the COGs, followed by “energy production and conversion”, and “cell wall/membrane/envelope biogenesis”, likely ensuring the continuation of metabolic processes in the host cell. Many of the virulence-associated factors found in the SaEVs (Table 1) were also included in the common pathogenic proteins of the *S. aureus* core EV proteome^27^.

The proteomic results suggest that SaEVs might play a critical role in modulating host cell functions by influencing its protein synthesis machinery, metabolism, and structural integrity. The identification of clusters related to peptidoglycan biosynthesis, antibiotic resistance, and stress response also proposes that SaEVs may play critical roles in maintaining bacterial integrity and adapting to host environments.

The presence of several key proteins, such as Hla, Clumping Factor B (ClfB), Enolase (eno) and Sortase A (srtA) suggested a role of SaEVs during *S. aureus*-host cell interaction and internalisation^27^. Additionally, the presence of other pathogenic proteins such as gamma-hemolysin and leukocidins (important for host cell lysis), Staphylococcal Accessory Regulator A (SarA) and phenol-soluble modulins (PSMs) (critical for biofilm formation), accessory gene regulator protein B (AgrB) (involved in quorum sensing), and fibrinogen- and elastin-binding proteins (essential for adhesion to host tissues) reinforces the theory of SaEVs as a crucial weapon to evade host defences and colonise the mammary gland.

The SaEV proteins may also influence host cytokine profiles in subclinical infections such as chronic mastitis. The upregulation observed in pro-inflammatory cytokines such as IL-1β and TNF-α could exacerbate the inflammation process, and a simultaneous upregulation of antiinflammatory IL-10 may suppress effective bacterial clearance, contributing to the tuned immune modulation characteristic of subclinical infections.

Effective vaccines need to be developed to minimise the use of antibiotics for prevention and to combat chronic intramammary infections that are resistant to treatment^4^. Bacterial EVs contain numerous pathogen-associated molecular patterns that can trigger both innate and adaptive immune responses, making them potential candidates for use as adjuvants and vaccines^42^. Adjuvants are anticipated to enhance the intensity, quality and duration of the adaptive immune response^43^. Our results show that SaEVs contain specific cell surface proteins and toxins that can elicit an effective innate and adaptive immune response. In particular, the upregulation of IL17A in boMdM and PBMCs is of great interest because it has been related to type 3 immunity, which has been proposed as a novel approach in *S. aureus* vaccination research^44^. Both SaEVs and non-EV secretions increased the expression of IL17A and INFG, but the response was higher in SaEVs. Thus, our findings endorse the potential of SaEVs as a promising strategy for developing vaccines against *S. aureus* infections.

In conclusion, our study emphasises the multifaceted roles of mastitis-related SaEVs in modulating host immune responses and contributing to *S. aureus* pathogenesis. Additionally, it lays the groundwork for future in vivo experiments into the molecular mechanisms of SaEV-mediated virulence and immune modulation. Finally, the proteomic profile of SaEVs provides a basis for unravelling the molecular components that underlie the observed morphological and gene expression changes and that could be targeted for novel therapeutic strategies such as developing SaEV-based vaccines.

## Materials and methods

### Isolation of *S. aureus* M5512B extracellular vesicles (SaEVs)

The M5512B *S. aureus* strain used in these experiments was kindly provided by Hans Ulrich Graber (Agroscope, Liebefeld, Switzerland) to the Laboratory of Food Microbiology collection at ETH Zurich (Zurich, Switzerland). *S. aureus* M5512B was grown in 1 L of TSB (Tryptic Soy Broth, Biolife) medium at 37 °C until the optical density at 600 nm (OD600) was 1.0. We obtained 9.96E + 07 Colony Forming Unit (CFU) per mL for this specific strain and culture. The culture medium was filtered with a 0.22 μm filter, and the filtrate was subjected to ultracentrifugation (UC).

All procedures before and after UC were performed under the sterile hood to avoid external contamination of SaEVs. The UC was performed in an Optimax 90XE ultracentrifuge (Beckman Coulter) with a 50.2 Ti rotor (Beckman Coulter). In total, 770 mL of *S. aureus* M5512B medium were divided into 20 tubes of 38.5 mL and centrifuged in two rounds at 150’000 x*g* for 2 h at 4 °C. The SaEV pellet of each tube was resuspended in 20 µL of sterile phosphate buffer saline PBS (Thermo Fisher, Gibco). The resuspended pellets from the same round were pooled in one sample and stored at -80 °C. Finally, 2 mL of the supernatant from each tube of one round were pooled in one sample and immediately subjected to additional ultracentrifugation (150’000 x*g* for 16 h at 4 °C) to obtain the SaEV-free supernatant (non-EV secretions).

### SaEV and non-EV secretions sterility test

The SaEVs and SaEV-free supernatant for cell stimulation were tested for environmental contamination due to the isolation procedure. For this, 50 µL of the samples were plated on an LB agar plate. Then, the plates were kept at 37 °C for 24 h to visualize the appearance of colonies.

### SaEV characterization

#### Transmission Electron microscope (TEM)

Transmission electron microscopy (TEM) was performed at the Scientific Center for Optical and Electron Microscopy service of ETH Zurich, as previously described^22^. Imaging of SaEVs and SN was done in a transmission electron microscopy (TEM) Morgagni 268 (Thermo Fisher) operated at 100 kV.

#### Tunable resistive pulse sensing (TRPS)

Particle size and concentration of isolated SaEVs were assessed using the qNano Gold system (Izon Science). More than 1000 particles were measured in an NP150 Nanopore using CPC100 beads as calibration particles. Analyses were performed with Izon Control Suite v.3.3.

#### Protein concentration measurement and Western blot (WB)

The SaEV protein concentration was determined using the Pierce™ BCA Protein Assay (Thermo Fisher) and a Nanodrop 2000 (Thermo Fisher). For western blots, 10 µg of SaEV sample from each round of ultracentrifugation were mixed with 10% beta-mercaptoethanol (Bio-Rad) and incubated for 5 min at 95 °C. Then, the samples were run in on an SDS-PAGE gel (Bio-Rad) for 35 min at 200 V. Proteins were transferred onto a 0.2 μm PVDF membrane in a Transturbo system (Bio-Rad) using 1.3 A, 25 V, and 7 min as transfer conditions. Blocking of the membrane was performed with 5% skim milk (Sigma-Aldrich) in Tris-buffered saline (TBS) with Tween^®^ 20 (TBS-T, 0.05% Tween^®^ 20) for 1 h. Membranes were incubated overnight with the Anti-α-hemolysin (Hla) primary antibody (ab190467, Abcam, 1:1000). After three washes of 5 min with TBS-T buffer, the membrane was incubated for 1 h with the secondary antibody goat anti-mouse IgG-HRP (sc2005, Santa Cruz Biotechnology,1:10’000). Precision Protein Strep Tactin-HRP was also added (1:10,000, Bio-Rad) to visualize the ladder. Following three more washes with TBS-T, an antibody signal was developed using the ClarityTM Western ECL kit (Bio-Rad), and images were acquired using the ChemiDoc MP Imaging System (Bio-Rad).

### Isolation and culturing of bovine monocyte-derived macrophages (boMdMs)

Peripheral blood mononuclear cells (PBMCs) were isolated from venous blood from 6 cows collected separately at a local slaughterhouse using 50 mL Leucosep tubes (Greiner bio one) filled with 15 ml separation medium (Histopaque^®^-1077, Sigma-Aldrich). Then, 8 mL of blood was mixed with the same amount of PBS and poured into the prepared Leucosep tubes. After centrifugation (10 min, 1000 xg, 21 °C, break off), the cell-enriched fraction with PBMCs was removed and washed twice with PBS. The remaining erythrocytes were lysed with 1 mL of ACK lysis buffer (Thermo Fisher, Gibco) and washed twice with PBS.

The boMdMs were generated from purified PBMCs using an adherence-based method^45,46^. Briefly, PBMCs were seeded in Nunc™ 4-Well Dishes (Thermo Fisher) in a complex medium (CM) consisting of RPMI-1640 with 20 mM 4-(2-hydroxyethyl)-1 piperazine ethane sulfonic acid (HEPES; Sigma-Aldrich), 10% heat-inactivated fetal bovine serum (FBS; Biochrom AG), 100 IU/mL penicillin and 100 g/mL streptomycin. To remove most non-adherent lymphocytes and non-viable cells, culture dishes were washed twice with sterile warm PBS after one hour of incubation (37 °C, 5% CO_2_). The complete medium was refreshed on day 3 and day 5 post-isolation. Cells stayed 7 days in culture until they developed into bovine monocyte-derived macrophages (boMdMs).

### Morphological characterization and CD14 immunostaining of boMdM

Morphological evaluation of boMdM was done using the DiffQuik staining (AMR Vet Collective), a commercial Romanowsky stain variant. The presence of boMdM CD14 + was confirmed by immunostaining with the anti-CD14 antibody (sc-1182, Santa Cruz Biotech). Briefly, boMdM cultured for 7 days were fixed with PFA 4% for 20 min and washed and stored in PBS at 4 °C until further used. Blocking was performed with filtered PBS complemented with 1% BSA for 1 h at room temperature (RT). Then, the primary antibody solution (dilution 1:100, in 0.1% BSA in PBS) or the non-primary antibody control solution (only 0.1% BSA in PBS) was added and incubated overnight in a cold room with gentle agitation. After 3 washes, the secondary antibody solution (anti-mouse Alexa 594, dilution 1:500), complemented with DAPI (dilution 1:500, D9542, Sigma-Aldrich), was added to the wells and incubated for 1 h at room temperature. Imaging of the boMdM was performed after three PBS washes with the confocal microscope Zeiss LSM 780 (Zeiss).

### Stimulation of boMdM and PBMCs with M5512B antigens

Six cultures of primary PBMCs and boMdM from six different cows were stimulated with 10 µg of M5512B SaEVs pool, M5512B non-EV secretions pool, and LTA (L2515, Sigma-Aldrich) prepared in 500 µL of CM medium. The control group (Ctr) received only CM, without M5512B *S. aureus* antigens. Following 24 h of incubation, the cells were rinsed twice with PBS, and 350 µL of RTL buffer (Qiagen) with 10% β-mercaptoethanol was added to each well to detach the cells. The cell mixture was then transferred to a tube and frozen at -80 °C for further analysis.

### RNA extraction and cDNA synthesis

RNA extraction was done using the Qiagen RNeasy^®^ Mini kit. The amount of RNA was measured with the Nanodrop 2000 (Thermo Fisher), and the integrity was evaluated using the BioAnalyzer 2100 (Agilent Technologies). Then, RNA was stored at -80 °C until further use. One hundred ng of total RNA was reverse transcribed using the RevertAid First Strand cDNA Synthesis Kit (Thermo Fisher).

### RT-qPCR and statistical analysis

The cDNA was diluted 1:10 in RNase-free water and combined with the master mix of the specific genes in the qPCR 384-well plate. The master mix was composed of 5 µL KAPA SYBR^®^ Fast (Sigma-Aldrich), 0.2 µL forward and reverse primer at concentrations of 10 µM and 0.6 µL of RNase-free water per sample. Each sample contained 6 µL of master mix and 4 µL of diluted cDNA. Per gene, one non-template control was run, plus two positive controls consisting of a pool of all the samples to test the pipetting error. Samples were run in a Biorad CFX ConnectTM device (Bio-Rad Laboratories Inc., Hercules, CA, USA). The program consisted of an initial denaturation of 15 min at 95 °C, followed by 40 cycles of 15 s at 95 °C and 30 s at 60 °C. The histone 3 mRNA expression was used as a reference for the relative quantification. The primers used in the RT-qPCR are listed in Supplementary Table 6. Gene expression analysis was performed using the following formula: relative expression (∆Cq) = (geometric average Cqreferences – (Cq target) + 20 ^23,47^. Statistical analysis was done using Graph Pad Prism software v9.0. First, values were tested for normality with the Shapiro-Wilk test. Then a repeated measures ANOVA or a mixed-effects model (when a value was missing) was used with Tukey’s multiple comparison test. Results with a *P* < 0.05 were considered statistically significant.

### ELISA analysis of interleukin 1 beta (IL1B)

Interleukin 1 beta (IL1B) levels were quantified using an enzyme-linked immunosorbent assay (ELISA) kit specific for bovine (ESS0027, Invitrogen, ThermoFisher). Briefly, 96-well plates were coated with the capture antibody specific for IL1B and incubated overnight at room temperature. Plates were washed to remove unbound antibodies and blocked with the blocking buffer (4% bovine albumin serum and 5% sucrose in PBS) for 1 h at RT. Diluted samples and standards were added to the wells and incubated for 1 h at RT. After washing, the detection antibody was added and incubated for 1 h at RT, followed by incubation with streptavidin-HRP conjugate for 30 min. Plates were developed with substrate solution, and the reaction was stopped with the stop solution (0.16 M sulfuric acid). Absorbance was measured at 450 and 550 nm. The IL1B concentrations were determined by comparing absorbance values to a standard curve and subtracting the 550 nm from the 450 nm values.

### SaEV and non-EV secretions proteomics

Proteomic analysis of SaEVs was analyzed by liquid chromatography coupled to high-resolution tandem mass spectrometry (LC-MS/MS) at the Functional Genomic Center Zurich (FGCZ). Briefly, samples were boiled for 10 min at 95 °C. Then, proteins were reduced by adding Tris(2-carboxyethyl)phosphine to a final concentration of 2 mM and alkylated by adding 2-chloroacetamide to a final concentration of 15 mM. After 30 min incubation at 30 °C (in the dark), samples were diluted with pure ethanol (EtOH) to reach a final concentration of 60% EtOH (v/v) before adding the corresponding amount of carboxylated magnetic beads (hydrophobic and hydrophilic) to the samples. After binding the proteins to the beads for 30 min at RT, beads were washed 3x with 80% EtOH. Enzymatic digestion was performed overnight at 37 °C using trypsin in 50 mM triethylammonium bicarbonate (TEAB) and adjusting the pH to 8. The supernatants were taken, and the remaining peptides were extracted from beads with water. The two elutions were combined and dried down. The digested samples were dissolved in aqueous 3% acetonitrile with 0.1% formic acid, and the peptide concentration was estimated with the Lunatic UV/Vis absorbance spectrometer (Unchained Lab). Peptides were separated on an M-class UPLC and analyzed on an Iontrap mass spectrometer (Thermo Fisher). The acquired MS data were processed using the Maxquant search engine (V 2.0.1.0^48^). The spectra were searched against the *Staphylococcus aureus* database NCTC 8325. The protein identification results were imported for further analysis in the software Scaffold v5.2.0 (Proteome Software Inc., Portland, OR).

### Proteomics data analysis

Proteins were filtered using a very stringent setting: protein FDR of 1%, a minimum of 2 peptides per protein, and a peptide FDR of 0.1%. Then, proteins identified as common contaminants at the FGCZ were also removed. A fold-change analysis was done using Scaffold v5.2.0. Functional enrichment analysis and MCL cluster analysis of protein-protein interaction were done using STRING v12.0 ^49^. Clusters of Orthologous Groups (COGs) were used to categorize SaEV proteins functionally^50^. To predict the protein locations, we used pSORTb v3.0.3 ^51^.

## Electronic supplementary material

Below is the link to the electronic supplementary material.

Supplementary Material 1

## Data Availability

The mass spectrometry proteomics data have been deposited in the ETH Research Collection (https://doi.org/10.3929/ethz-b-000706440). Further inquiries can be directed to the corresponding author.
